# Efficacy and Safety of Tirzepatide in Patients with Type 2 Diabetes Mellitus: A Systematic Review and Meta-Analysis of Randomized Phase II/III Trials

**DOI:** 10.3390/ph14100991

**Published:** 2021-09-28

**Authors:** Akshaya Srikanth Bhagavathula, Kota Vidyasagar, Wubshet Tesfaye

**Affiliations:** 1 Department of Social and Clinical Pharmacy, Faculty of Pharmacy in Hradec Králové, Charles University, 50115 Hradec Králové, Czech Republic; 2 University College of Pharmaceutical Sciences, Kakatiya University, Warangal 506009, India; vidyasagarkota2@gmail.com; 3 Health Research Institute, University of Canberra, Canberra, ACT 2617, Australia; wubshet.tesfaye@canberra.edu.au

**Keywords:** LY3298176, tirzepatide, diabetes, glucose, randomized controlled trial, efficacy

## Abstract

Tirzepatide is a novel once-a-week dual glucose-dependent insulinotropic polypeptide and glucagon-like peptide-1 receptor agonist, currently under trial to assess glycemic efficacy and safety in people with type 2 diabetes. A systematic review and meta-analysis were conducted to investigate the efficacy of tirzepatide on glycated hemoglobin (HbA1c, %), fasting serum glucose (mg/dL), and body weight (kg) in patients with uncontrolled type 2 diabetes (HbA1c > 7.0%). Mean changes for efficacy and proportions (safety) with corresponding 95% confidence intervals (CIs) were used to provide pooled estimates. A total of four randomized controlled trials, comprising 2783 patients of whom 69.4% (n = 1934) were treated with 5 mg (n = 646), 10 mg (n = 641), or 15 mg (n = 647) of tirzepatide, were compared to the placebo (n = 192) or the selective GLP-1 receptor agonist (n = 523). The pooled analysis showed that tirzepatide treatment resulted in a greater lowering of the HbA1c (−1.94%, 95% CI: −2.02 to −1.87), fasting serum glucose (−54.72 mg/dL, 95% CI: −62.05 to −47.39), and body weight (−8.47, 95% CI: −9.66 to −7.27). We also found that improvement in the HbA1c levels was still maintained at weeks 26 and 40 from the long-term trials. As for safety, only 3% experienced hypoglycemia, and 4% (95% CI: 2 to 6) experienced serious adverse events, while the discontinuation of therapy percentage was 7% (95% CI: 5 to 8). Tirzepatide significantly improved glycemic control and body weight and had an acceptable safety profile, indicating that it is an effective therapeutic option for glucose-lowering in patients with type 2 diabetes mellitus.

## 1. Introduction

Diabetes mellitus affects over 463 million people globally as of 2019, with this number projected to grow to 700 million by 2045 [1]. The well-recognized microvascular and macrovascular damages associated with type 2 diabetes mellitus (T2DM) make it among the leading causes of serious complications, including blindness, kidney failure, and cardiovascular complications, and associated mortality [2,3]. Optimal management of T2DM is required to effectively prevent the occurrence of diabetes-related complications or delay the deterioration of these complications if they are already present [4]. A range of drug classes with different modes of action have been in use for the management of diabetes and/or its major clinical sequelae.

While the various classes of T2DM medications have their own benefits, ideal treatments would be those that are effective in reducing blood glucose levels, are associated with less risk of hypoglycemia and weight gain, and have added benefits for cardiovascular or renal outcomes [5]. Some of the newly emerging treatments used for T2DM, such as glucagon-like peptide-1 (GLP-1) receptor agonists and the sodium-glucose cotransporter-2 inhibitors, have, for example, showed promising outcomes in recent years in terms of providing additional cardiorenal benefits in addition to controlling diabetes [6].

GLP-1 agonists, which include short-acting or long-acting agents, have been associated with improved glycemic control and cardiovascular outcomes, as well as body weight reduction in people with T2DM [7]. The other incretin hormone, glucose-dependent insulinotropic polypeptide (GIP), despite its similarity with GLP-1 and its receptors, is devoid of effects like the inhibition of appetite and food intake, which is seen with the use of GLP-1 [8]. Therefore, combining the GLP-1 agonists with other agents that act on glucose-dependent insulinotropic polypeptide (GIP) receptors has been hypothesized to produce a more effective control of glycemia along with a significant weight reduction effect [9]. Tirzepatide is a novel antidiabetic treatment recently developed and has demonstrated favorable body weight, cardiovascular, and renal-related outcomes [10]. It acts as an agonist of both GLP-1 and GIP receptors, rendering it with an additive effect of producing a more pronounced insulin response than can be achieved through individual hormone administration [5,11,12].

Early evidence revealed that tirzepatide is associated with a significantly positive outcome in controlling both blood glucose and body weight [8]. In light of additional emerging evidence based on phase II and III clinical trials investigating the effect of tirzepatide on glycemic and other added benefits, there is a need for more comprehensive data to consolidate the evidence and guide future research and potential clinical use. Therefore, this work aims to present a systematic review and meta-analysis of the literature published on phase II and III trials involving tirzepatide with a particular focus on its link with glycemic and body weight parameters.

## 2. Materials and Methods

### 2.1. Search Strategy

The systematic review and meta-analysis were executed in full accordance with the preferred reporting items for systematic review and meta-analyses guidelines [13]. A comprehensive literature search was conducted in the PubMed/Medline, Cochrane Library, Web of Science, and Scopus databases, which were searched for randomized controlled trials (RCTs) investigating the efficacy and safety of tirzepatide up to 30 July 2021 and published in the English language. The following medical subject heading terms and keywords were used for the database searches: (tirzepatide OR LY3298176 OR LY-3298176) AND (diabetes mellitus OR diabetes OR type 2 diabetes mellitus OR hyperglycaemia OR hyperglycemia OR hyperglycaemias OR hyperglycemias OR hyperglycemia s OR glucose OR glycated hemoglobin AND body weight). We also hand-searched the databases to identify additional relevant studies, references of relevant review articles, and conference proceedings.

### 2.2. Study Selection

Original studies that met the following criteria were included: (i) population: patients with uncontrolled T2DM, with no restriction on complications; (ii) were a phase II or III RCT with either a single-center or multicenter design; (iii) investigating the effect of tirzepatide on glucose parameters or body weight; (iv) intervention: tirzepatide at all doses (5 mg, 10 mg, 15 mg); (v) control: placebo or control; (vi) outcomes: efficacy and safety parameters related to the treatment; (vii) tested the efficacy and safety of tirzepatide in the short (12 weeks) and long term (26 and 40 weeks); and (viii) safety outcomes included all adverse events (AEs), hypoglycemic events, gastrointestinal events, and discontinuation of therapy.

The exclusion criteria included: (a) nonhuman studies and in vitro research, phase I clinical trials, case reports, editorials, conference proceedings, commentaries, expert opinions, reviews, studies without original data, non-RCTs, non-English publications, and duplicate publications; and (b) studies that lacked a control-treated group for comparison with tirzepatide.

### 2.3. Data Extraction

Two authors independently performed the literature search. After the identification of eligible studies, the authors independently extracted data using standardized predefined forms for the first author’s name; clinical trial registration number; year of publication; study sites; study design; baseline characteristics of the study population; age; the number of males; population; groups; duration of disease; baseline HbA1c; background glucose-lowering therapy; tirzepatide and comparator(s); dose/duration of tirzepatide; and outcomes measured. We extracted outcome data as arm-specific counts (i.e., number of participants, means differences, and standard error (or standard deviation) for continuous outcomes in patients with T2DM).

We also extracted data at the corresponding time point to appraise the HbA1c efficacy of tirzepatide in the short (week 12) and long term (weeks 26 and 40). Among the identified studies, the primary efficacy endpoint was the mean changes in HbA1c, fasting serum glucose (FSG, mg/dL), and body weight (kg) with tirzepatide at doses of 5 mg, 10 mg, and 15 mg monotherapy or add-on therapy to metformin in patients with T2DM. With regard to safety, we considered AEs, serious AEs, hypoglycemic events, gastrointestinal events, and AEs leading to discontinuation of treatment.

### 2.4. Quality Assessment

The risk of bias in the eligible RCTs was performed using the “Cochrane collaboration’s tools for quality assessment of randomized controlled trials” [14]. The following domains were considered: (i) selection bias: random sequence generation and allocation concealment; (ii) blinding of outcome assessment (detection bias); (iii) incomplete outcome bias (attrition bias); (iv) selective reporting (reporting bias); and other bias (e.g., whether the baseline was comparable).

### 2.5. Data Analysis

All the analyses were performed using STATA MP 16.1 software (StataCorp LLC, College Station, TX, USA). Changes in continuous outcomes were calculated for each study arm by subtracting the value at baseline from the value after intervention. All the efficacy estimates were expressed as mean changes and 95% confidence interval (CI) from baseline. Standard deviations (SD) were calculated from the standard error or 95% CI, according to the Cochrane handbook for systematic review of interventions [15]. Safety estimates were presented as a pooled proportion with 95% CI.

The Higgins *I^2^* statistics and Cochran’s Q test were used to assess the potential statistical heterogeneity among trials. The meta-analysis was conducted using a fixed-effect model (using inverse-variance) or a random-effect model (DerSimonian–Laird method) based on low heterogeneity (<50%) or high heterogeneity (>50%). Publication bias was evaluated using funnel plots, Egger’s regression asymmetry test, and Begg’s correlation rank test [16]. A two-tailed *p*-value was considered significant below the level of 0.05.

## 3. Results

### 3.1. Study Characteristics

**Figure 1:**
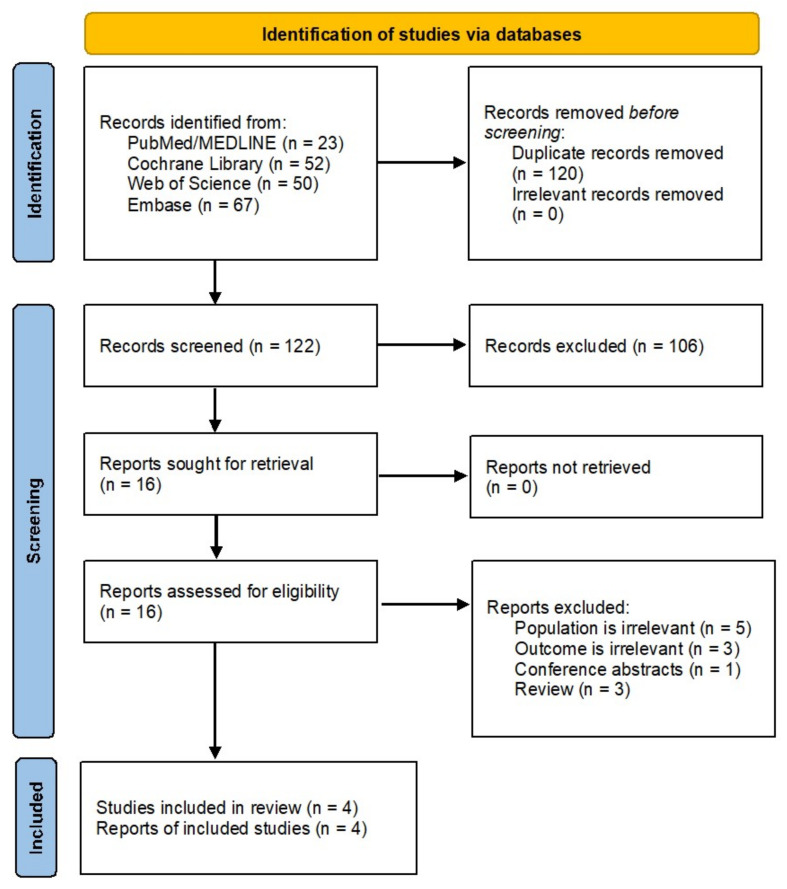
PRISMA flowchart of literature search and study selection.

From 242 identified records, we excluded nonhuman and irrelevant studies, leaving 16 reports for full-text assessment. After further selection (Figure 1), four unique RCTs fulfilled the inclusion criteria (Table 1) [17,18,19,20]. RCTs published between 2018 and 2021 included 2783 (range: 26–1490) participants with T2DM; three out of four studies were multinational RCTs. All the studies included T2DM patients controlled inadequately with diet and exercise alone or with stable metformin therapy. Baseline age and disease duration weighted means were 56.4 ± 1.82 and 8 years, respectively, 53% of the participants were male, and follow-up duration ranged from 12 to 40 weeks. Other characteristics of the RCTs, such as study details, drug- and outcome-specific data, are shown in Table 1.

**Figure 2:**
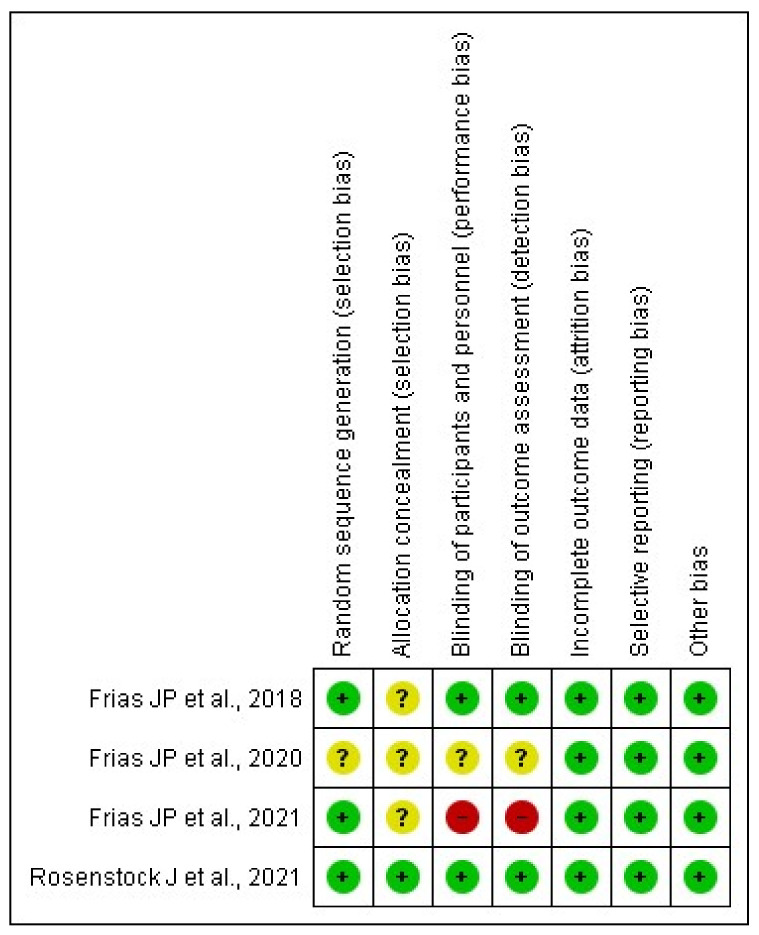
Assessment of the risk of bias in included studies with Cochrane domain-based quality assessment tool.

Overall, the risk of bias in eligible RCTs is shown in Figure 2. All included studies exhibit a low risk of selection bias, performance bias, detection bias, and reporting bias. The risk of bias in allocation concealment was generally unclear apart from the Rosenstock et al. [17] study. One study [20] has a high risk of bias because it did not provide details of blinding the participants and their outcome assessment.

### 3.2. Meta-Analyses

#### 3.2.1. Efficacy of Tirzepatide on Glycated Hemoglobin

**Figure 3:**
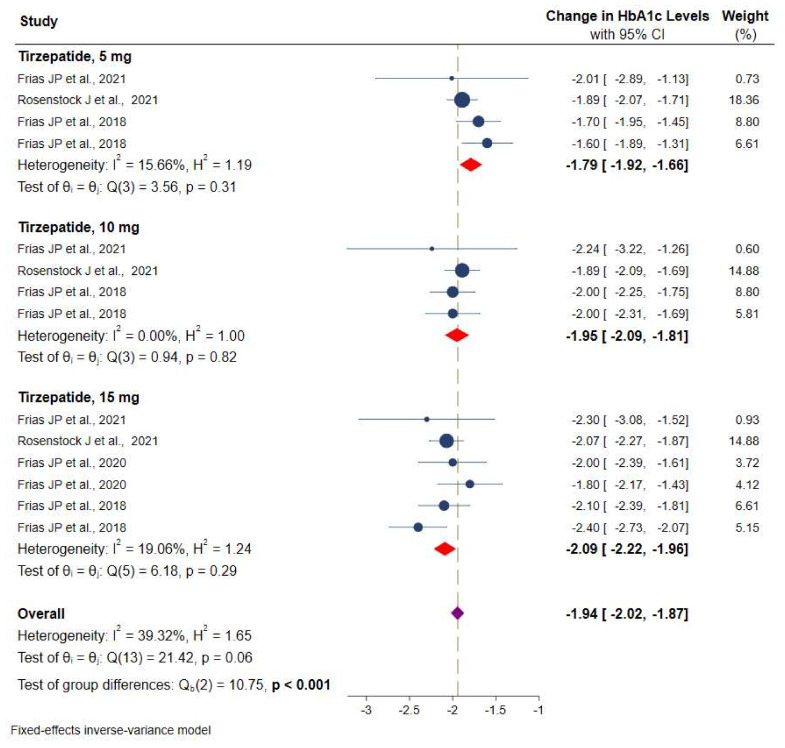
Effect of once-weekly tirzepatide on glycated hemoglobin (HbA1c).

Data on HbA1c were available from all RCTs. Figure 3 shows the efficacy of different doses of tirzepatide monotherapy or add-on to metformin on HbA1c using a fixed-effects model. The included studies displayed varying degrees of glycemic efficacy. Overall, meta-analyses showed a significant reduction in HbA1c versus placebo/controls by −1.9% (95% CI: −2.0 to −1.8, *p* < 0.001; *I^2^* = 39.3%), from −1.8% (95% CI: −1.9 to −1.6) for tirzepatide 5 mg, −1.9% (95% CI: −2.1 to −1.8) for 10 mg, and −2.1% (95% CI: −2.2 to −1.9) for 15 mg, respectively.

**Figure 4:**
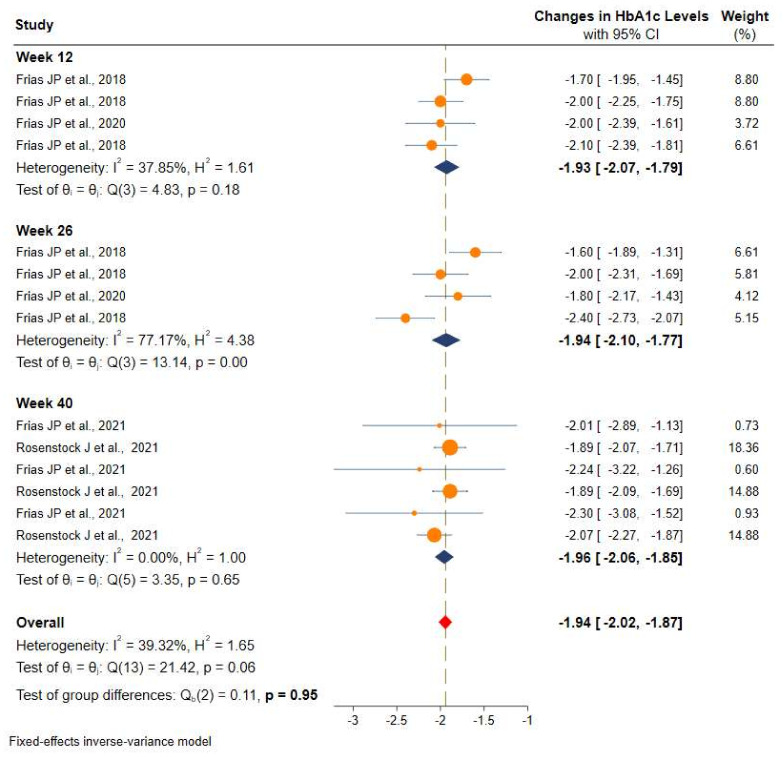
Efficacy of once-weekly tirzepatide on glycated hemoglobin (HbA1c) based on duration of intervention.

Studies were stratified based on the duration of intervention and showed a more significant and consistent reduction in the HbA1c after 12 weeks (−1.9% (95% CI: −2.0 to −1.8), 26 weeks (−1.9%, 95% CI: −2.1 to −1.7), and 40 weeks (−1.9% (95% CI: −2.0 to −1.8) of intervention with tirzepatide than the placebo or controls. More details can be found in Figure 4.

#### 3.2.2. Efficacy of Tirzepatide on Fasting Serum Glucose

**Figure 5:**
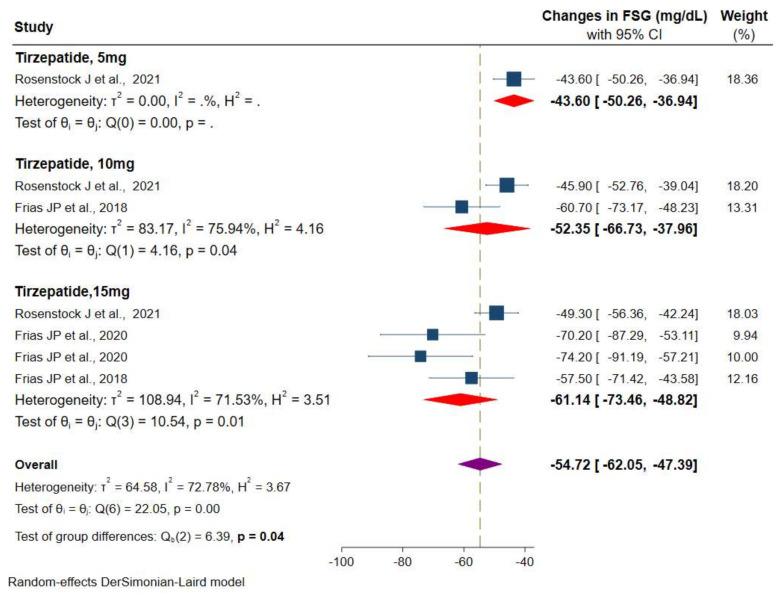
Effect of once-weekly tirzepatide on fasting serum glucose levels (mg/dL).

Values of FSG were available from three RCTs. The meta-analysis of available data showed a significant reduction in FSG of −54.7 mg/dL (95% CI: −62.0 to −47.4) versus placebo/controls for three different doses of the tirzepatide intervention group, from −43.6 mg/dL (95% CI: −50.2 to −36.9) for tirzepatide 5 mg, −52.3 mg/dL (95% CI: −66.7 to −37.9) for tirzepatide 10 mg, and superior reduction in tirzepatide 15 mg, −61.1 mg/dL (95% CI: −73.4 to −48.8) was observed (Figure 5). However, the heterogeneity among these studies was statistically significant (*I*^2^ = 72.7%, *p* < 0.001).

#### 3.2.3. Effect of Tirzepatide on Body Weight

**Figure 6:**
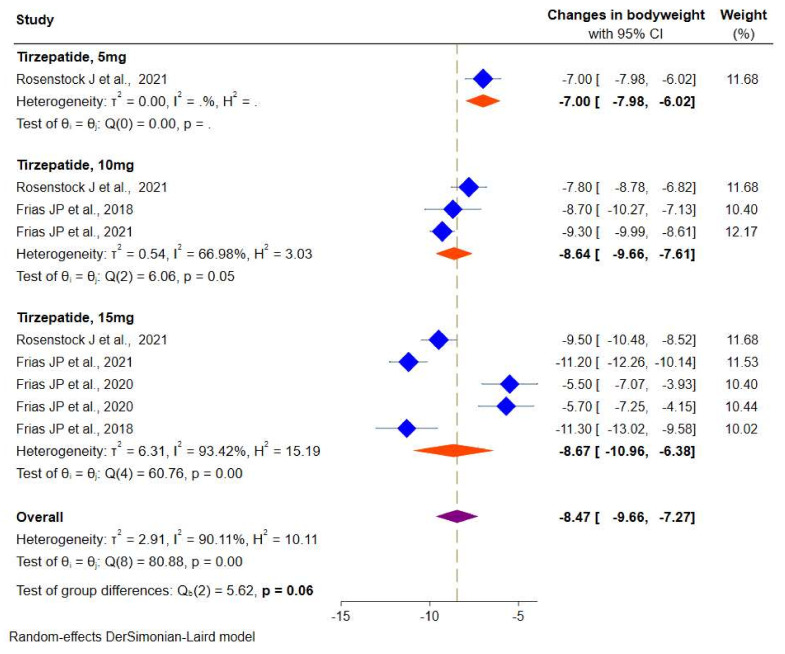
Effect of once-weekly tirzepatide on body weight (kg).

Data on body weight were available from four RCTs. Random-effects meta-analyses showed a significant reduction in body weight in the tirzepatide group versus the placebo/controls by −8.4 kg (95% CI: −9.6 to −7.2), from −7.0 kg (95% CI: −7.9 to −6.0) for tirzepatide 5 mg, −8.6 kg (95% CI: −9.6 to −7.6) for tirzepatide 10 mg and 15 mg (−8.6 kg, 95% CI: −10.9 to −6.3), respectively (Figure 6). There was a considerable heterogeneity among the studies (*I*^2^ = 90.1%, *p* < 0.001).

#### 3.2.4. Overall Safety

Table 2 shows the proportion of representative adverse events in the tirzepatide-treated group. All the studies provided data on the safety outcomes. The overall pooled proportion of hypoglycemic events was 3% (95% CI: 2–5), 4% (95% CI: 2–6) for serious AEs, and AEs leading to discontinuation of therapy were 7% (95% CI: 5–8). A significantly higher proportion of patients experienced gastrointestinal adverse events with tirzepatide (44%, 95% CI: 40–48).

#### 3.2.5. Publication Bias

**Figure 7:**
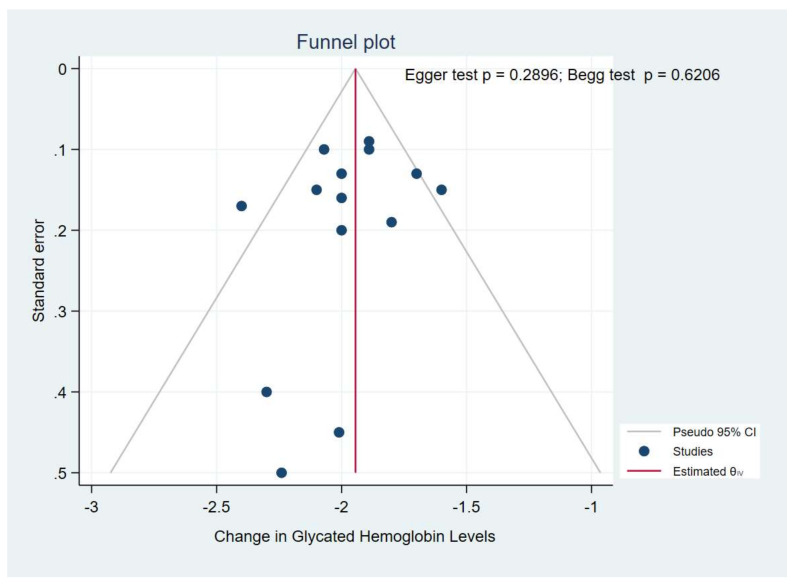
Funnel plot.

A visual inspection of funnel plots for the primary outcome (HbA1c) suggested an absence of publication bias and of a small study effect, the Egger’s test (*p* = 0.289) and Begg’s test (*p* = 0.620) indicated no evidence of publication bias (Figure 7). Because a publication bias assessment was not recommended for less than 10 pooled studies, we did not assess the existence of publication on FSG and body weight.

## 4. Discussion

T2DM is a chronic disease characterized by hyperglycemia and progressive dysregulation of the insulin-glucose feedback mechanism, and glucose-lowering is a mainstay of treatment [21]. Novel glucose-lowering drugs such as selective GLP-1 receptor agonists, especially once-weekly dulaglutide and semaglutide, showed a substantial improvement in glucose control and promoted weight loss [22]. Despite these advances in therapy for the management of hyperglycemia, many people with T2DM did not achieve optimal glycemic control. Notably, the co-administration of the GLP-1 receptor agonist and GIP in mice with T2DM and obesity showed a greater improvement in glycemic and body weight than with selective GLP-1 receptor agonists [23]. This rationale paved the way to target both incretin hormones and the development of a dual GLP-1/GIP receptor agonist, referred to as a “twincretin” for the treatment of T2DM [9]. Tirzepatide is a novel therapeutic agent administered once a week as a dual GLP-1/GIP receptor, formulated as a 39-amino acid synthetic peptide, based on the native GIP sequence [9]. In individual RCTs, tirzepatide alone or added to metformin was shown to improve glycemic control with weight loss compared with a placebo or selective GLP-1 receptor agonists (dulaglutide or semaglutide) [17,18,19,20]. However, to date, no systematic compilation of the available evidence of tirzepatide has been performed.

We presented the relative efficacy and safety of tirzepatide monotherapy or add-on to metformin compared with the placebo or selective GLP-1 receptor agonists to provide a comprehensive analysis of the tirzepatide. Compared to the placebo or the selective GLP-1 receptor agonists, tirzepatide improved glycemic control (−2.0 to −1.8% in HbA1c and −62.0 to −47.3 mg/dL in FSG) and reduced body weight (−9.6 to −7.2 kg). All three tirzepatide doses showed significant HbA1c reductions, and the robust glycemic effect was consistently maintained from week 12 to week 40. Available evidence from the published RCTs also suggested a greater decrease in HbA1c, FSG, and body weight [19,20,21,22]. Given the limited data available for postprandial self-monitored blood glucose profile, however, the effect of tirzepatide on the achievement of the guidelines’ recommended HbA1c targets and weight loss of 5% or greater should be further investigated. Overall, the highest dose of tirzepatide had superior effects on HbA1c reduction, and the change in the HbA1c presumably occurred as early as 12 weeks, with no differences noted at week 26 or 40. The most recent phase 3 trial (SURPASS-1) has indeed demonstrated a similar reduction in HbA1c, FSG, and body weight in patients with T2DM treated with tirzepatide (5, 10, and 15 mg) as monotherapy [17]. Ongoing RCTs [24,25,26,27,28,29] will confirm whether similar benefits could be expected in an overweight or obese population with (SURMOUNT-2) [29] or without T2DM (SURMOUNT-3, SURMOUNT-4) [25,26] and in patients with T2DM inadequately controlled on insulin glargine with/without metformin (SURPASS-6) [27]. Moreover, a recently completed phase 1 trial on tirzepatide (NCT03951753) may provide further evidence on the relationship between HbA1c and body weight reduction in patients with T2DM [28].

Along with changes in HbA1c, we also found some marginal differences with tirzepatide doses on FSG and body weight. The highest dose of tirzepatide (15 mg) reduced FSG to a greater extent than other doses (5 mg and 10 mg). Conversely, tirzepatide 10 mg and 15 mg did not differ in the extent of body weight reduction. Little data on FSG and body weight with different doses of tirzepatide are reported in the included RCTs, limiting the ability to investigate clinically meaningful differences between higher and lower doses and also accounting for different background therapies. Indeed, the differences observed for some clinical outcomes could be attributed to the intrinsic pharmacological properties of investigational drugs at different doses.

As for safety, tirzepatide therapy did not lead to a higher incidence of all AEs than the placebo or selective GLP-1 receptor agonists, and individual studies indicated that most AEs were mild or moderate in severity [17,18,19,20]. The most prevalent AEs were gastrointestinal (44%), including nausea and diarrhea, and similar AEs observed in current GLP-1 analogs [30]. Moreover, a limited proportion of participants experienced hypoglycemia (3%) and AEs leading to the discontinuation of tirzepatide (7%), most of which are dose-dependent incidences. Ongoing RCTs [5,6,7,9] will confirm whether tirzepatide exhibits long-term safety, and SURPASS-CVOT (NCT04255433) [31] will provide more data on the cardiovascular safety of tirzepatide compared with dulaglutide.

To the best of our knowledge, this is the first systematic review and meta-analysis that combined the data of all tirzepatide phase 2 and phase 3 RCTs with a mixed-treatment comparison meta-analytical approach aiming to provide a comprehensive picture of clinically meaningful efficacy and safety outcomes. However, we should acknowledge some limitations of this study. We identified a small number of studies and performed a meta-analysis based only on data published in journal articles or available at ClinicalTrials.gov. Given the limited available data, we pooled efficacy data for HbA1c, FSG, and body weight and could not perform a subgroup analysis to investigate the source of heterogeneity for some outcomes. However, RCTs, ethnic differences in the population, differences in the settings, follow-up duration or outcome selections, definitions, and ascertainment could differ. Moreover, we found a data inconsistency for the FSG and body weight meta-analysis, and caution is recommended in interpreting these results. Lastly, the current duration of studies was very limited, and data about tirzepatide in the long term were deficient.

## 5. Conclusions

Tirzepatide, a novel incretin-based therapy for T2DM, showed robust HbA1c (−1.94%), FSG (−54.7 mg/dl), and body weight (−8.5 kg) reductions, without an increased risk of hypoglycemia. At the highest dose (15 mg), tirzepatide reduced: HbA1c (−2.1%), FSG (−61.1 mg/dl), and body weight (−8.6 kg). The study also found that tirzepatide had an acceptable safety profile similar to the GLP-1 receptor agonists for the treatment of T2DM. Further research is needed to determine the long-term safety and efficacy of tirzepatide in achieving guideline-recommended cardiometabolic targets.

## Figures and Tables

**Table 1:** Study characteristics of included studies.

Author Name	NCTID	Location	Design	Participants Condition at Baseline	Disease Duration (years)	Primary Outcome	Treatment Duration (weeks)	Intervention	Patients on Metformin Therapy (%)	Number Randomized	Age (Years)	Male (%)	Average Change in HbA1c (%) From Baseline
Rosenstock J et al., 2021 [17]	NCT03954834 (SURPASS-1)	India, Japan, Mexico, and USA	Multicenter, double-blind, randomized, placebo-controlled, phase 3 trial	T2DM ([HbA1c] 7.0–9.5%) that was inadequately controlled with diet and exercise alone. They were naive to injectable diabetes therapy.	4.7	Change in HbA1c	40	Tirzepatide 5 mg/day	NR	121	54.1	46	−1.87
								Tirzepatide 10 mg/day	NR	121	55.8	60	−1.89
								Tirzepatide 15 mg/day	NR	121	52.9	52	−2.07
								Placebo	NR	115	53.6	49	0.04
Frias JP et al., 2018 [18]	NCT03131687	Poland, Puerto Rico, Slovakia, and USA	Multicenter, phase 2b, randomized, double-blind study	T2DM for at least 6 months ([HbA1c] 7.0–10.5%) that was inadequately controlled with diet and exercise alone or with stable metformin therapy for at least 3 months before screening.	9.0	Change in HbA1c	26	Tirzepatide 1 mg/day	88.5	52	57.4	56	−0.7
								Tirzepatide 5 mg/day	89.1	55	57.9	62	−1.6
								Tirzepatide 10 mg/day	86.3	51	56.5	59	−2.0
								Tirzepatide 15 mg/day	96.2	53	56.0	42	−2.4
								Dulaglutide 1.5 mg/day	88.1	54	58.7	44	−1.1
								Placebo	92.2	51	56.6	57	0.1
Frias JP et al., 2020 [19]	NCT03311724 (SURPASS)	USA	Multicenter, phase 2, randomized, double-blind, placebo-controlled	T2DM for at least 6 months ([HbA1c] 7.0–10.5%) that was inadequately controlled with diet and exercise alone or with stable metformin therapy.	9.1	Change in HbA1c	12	Tirzepatide 12 mg/day	86.2	29	61.2	51.7	−1.7
								Tirzepatide 15 mg/day-1	89.3	28	55.5	57.1	−2
								Tirzepatide 15 mg/day-2	82.1	28	56.6	82.1	−1.8
								Placebo	88.5	26	56	46.2	0.2
Frias JP et al., 2021 [20]	NCT03987919 (SURPASS-2)	USA, UK, Argentina, Australia, Brazil, Canada, Israel, Mexico	Multicenter, phase 3, open-label, parallel-group, randomized, active-controlled	T2DM for at least 6 months ([HbA1c] 7.0–10.5%) that was inadequately controlled with metformin therapy.	8.6	Change in HbA1c	40	Tirzepatide 5 mg/day	100	470	56.3	43.6	−2.01
								Tirzepatide 10 mg/day	100	469	57.2	50.7	−2.24
								Tirzepatide 15 mg/day	100	470	55.9	45.5	−2.3
								Semaglutide 1 mg/day	100	469	56.9	48	−1.86

NR: Not reported; HbA1c: Glycated hemoglobin; T2DM: Type 2 diabetes mellitus; UK: United Kingdom; USA: United States of America.

**Table 2:** Safety of tirzepatide.

Events	Proportion (95% CI)				*I* ^2^	*P*_hetetogeneity_
	Total	Tirzepatide, 5 mg	Tirzepatide, 10 mg	Tirzepatide, 15 mg		
Total adverse events	70 (67–74)	66 (61–71)	70 (65–75)	73 (65–81)	58.5%	0.305
Serious adverse events	4 (2–6)	5 (1–8)	4 (1–7)	3 (0–7)	73.2%	0.071
Hypoglycaemia	3 (2–5)	4 (1–8)	5 (1–11)	7 (2–13)	80.1%	0.594
All gastrointestinal events	44 (40–48)	39 (35–43)	46 (42–49)	50 (38–61)	63.6%	0.025
Nausea	5 (2–7)	1 (0–2)	3 (0–7)	12 (6–18)	89.9%	<0.001
Diarrhea	3 (2–5)	0 (0–1)	3 (1–8)	13 (6–19)	89.9%	<0.001
Adverse events leading to discontinuation of therapy	7 (5–8)	5 (3–8)	7 (5–10)	8 (4–12)	51.7%	0.441

## Data Availability

Data sharing not applicable.
